# Supplementary material for: Evaluating active versus passive sources of human brucellosis in Jining City, China
Source: PeerJ. 2021 Jun 22;9:e11637. doi: 10.7717/peerj.11637 (PMC8231335; doi:10.7717/peerj.11637)
Supplement: Supplemental Information 1 [file peerj-09-11637-s001.docx]

Supplemental Table 1. Self-designed Questionnaire.

Self-designed Questionnaire on human brucellosis in Jining city

1. ID

2. age(year):

3.Sex: a. Male b. Female

4.Ethnicity: a. Han b. Hui c. other

5. Education:

a. Under junior middle school

b. Junior middle school

c. Above junior middle school

6.Career: a. Famer b. Worker c. Children d. Student e. Veterinarian f. Other

7.Do you have contact history of sheep or cow: a. Yes b. No,

if yes, please go on: Which kind of the contact?

a. Cultivation

b. Slaughter

c. Selling

d. Process

e. Other

8.Clinic syndrome (please check all answers):

a. Fever b. Arthralgia c. Debilitation d. Hyperhidrosis e. Muscle pain f. Other

9.Source Group: a. Active detection group b. Passive detection group.

IF passive group, please go on: a. From hospital b. From CDC

10.HB Result: a. Positive b. Neglect

11. Please list your contact TEL:
