# Supplementary material for: Evaluating active versus passive sources of human brucellosis in Jining City, China
Source: PeerJ. 2021 Jun 22;9:e11637. doi: 10.7717/peerj.11637 (PMC8231335; doi:10.7717/peerj.11637)
Supplement: Supplemental Information 2 [file peerj-09-11637-s002.docx]

**Supplemental Table 2. The associated factors related to the positive detection of** **human** **brucellosis in passive detection group from hospital.**

| Characteristics | Passive detection group from hospital | |  |
| --- | --- | --- | --- |
|  | OR (95% CI) | P |  |
| Age, year |  |  |  |
| ≤45 | Reference |  |  |
| 45~60 | 0.43 (0.16-1.18) | 0.103 |  |
| >60 | 0.80 (0.28-2.30) | 0.682 |  |
| Sex |  |  |  |
| Male | 1.57 (0.64-3.81) | 0.323 |  |
| Female | Reference |  |  |
| Ethnicity |  |  |  |
| Han | 0.78 (0.06-10.4) | 0.849 |  |
| Hui | Reference |  |  |
| Education |  |  |  |
| Under junior middle school | Reference |  |  |
| Junior middle school | 1.17 (0.48-2.86) | 0.735 |  |
| Above junior middle school | 0.84 (0.21-3.4) | 0.809 |  |
| Career |  |  |  |
| Worker | Reference |  |  |
| Farmer | 1.26 (0.35-4.58) | 0.723 |  |
| Children/Student | 0.69 (0.05-9.26) | 0.782 |  |
| Veterinarian | - | - |  |
| Other | 1.77 (0.43-7.19) | 0.427 |  |
| Contact history of sheep or cow |  |  |  |
| Cultivation | 7.97 (3.12-20.35) | <0.001 |  |
| Slaughter | 2.10 (0.34-13.12) | 0.426 |  |
| Selling | 8.13 (0.64-103.03) | 0.106 |  |
| Process | 6.15 (0.99-37.81) | 0.051 |  |
| Clinical manifestation |  |  |  |
| Fever | 0.94 (0.36-2.42) | 0.894 |  |
| Arthralgia | 2.16 (0.77-6.05) | 0.143 |  |
| Debilitation | 0.75 (0.23-2.44) | 0.629 |  |
| Hyperhidrosis | 5.46 (2.23-13.40) | <0.001 |  |
| Muscle pain | 1.42 (0.56-3.60) | 0.459 |  |
| Other | 0.98 (0.25-3.77) | 0.973 |  |

OR, odds ratio; CI, confidence interval.
