# Supplementary material for: Evaluating active versus passive sources of human brucellosis in Jining City, China
Source: PeerJ. 2021 Jun 22;9:e11637. doi: 10.7717/peerj.11637 (PMC8231335; doi:10.7717/peerj.11637)
Supplement: Supplemental Information 4 [file peerj-09-11637-s004.docx]

附. 中文版调查问卷

济宁市布病监测调查表

1. 编号

2. 年龄（实足）：

3. 性别：①男 ②女

4. 民族：①汉族 ②回族 ③其他

5. 文化程度：

①初中以下

②初中

③初中以上

6. 职业：①农民 ②工人 ③散居儿童 ④学生 ⑤兽医 ⑥其他

7. 你是否曾经有过牛、羊接触史：①是 ②否

若‘是’请继续回答以下问题：通过何种途径接触？

①养殖牛羊

②屠宰牛羊

③贩卖牛羊或经营牛羊制品

④加工牛羊制品

⑤其他

8. 您现在的症状是(多选)①发热：最高 度,持续 天 ②关节痛部位: ③乏力④多汗⑤肌肉疼⑥其他

9. 您是如何发现布病的？①主动检查 ②被动检查

若‘被动检查’，地点为以下哪个？①医院（级别 ）②疾控中心

10. 布病检测结果：①阳性 ②阴性

11. 请附上您的联系方式：
